# Supplementary material for: Food Accessibility and Nutritional Outcomes Among Food-Insecure Pregnant Women in Singapore
Source: Nutrients. 2025 Feb 27;17(5):835. doi: 10.3390/nu17050835 (PMC11902187; doi:10.3390/nu17050835)
Supplement: Supplementary file 1 [file nutrients-17-00835-s001.zip › nutrients-3465574-supplementary.pdf]

# Checklist S1: COnsolidated criteria for REporting Qualitative research (COREQ) checklist

| Topic | Item No. | Guide Questions/Description | Reported on Page No. |
|---|---|---|---|
| **Domain 1: Research team and reflexivity** | | | |
| *Personal characteristics* | | | |
| Interviewer/facilitator | 1 | Which author/s conducted the interview or focus group? | 4 |
| Credentials | 2 | What were the researcher's credentials? E.g. PhD, MD | 4 |
| Occupation | 3 | What was their occupation at the time of the study? | 4 |
| Gender | 4 | Was the researcher male or female? | 4 |
| Experience and training | 5 | What experience or training did the researcher have? | 4 |
| *Relationship with participants* | | | |
| Relationship established | 6 | Was a relationship established prior to study commencement? | 4 |
| Participant knowledge of the interviewer | 7 | What did the participants know about the researcher? e.g. personal goals, reasons for doing the research | 4 |
| Interviewer characteristics | 8 | What characteristics were reported about the inter viewer/facilitator? e.g. Bias, assumptions, reasons and interests in the research topic | 4 |
| **Domain 2: Study design** | | | |
| *Theoretical framework* | | | |
| Methodological orientation and Theory | 9 | What methodological orientation was stated to underpin the study? e.g. grounded theory, discourse analysis, ethnography, phenomenology, content analysis | 3 |
| *Participant selection* | | | |
| Sampling | 10 | How were participants selected? e.g. purposive, convenience, consecutive, snowball | 2 to 3 |
| Method of approach | 11 | How were participants approached? e.g. face-to-face, telephone, mail, email | 2 |
| Sample size | 12 | How many participants were in the study? | 5 |
| Non-participation | 13 | How many people refused to participate or dropped out? Reasons? | N/A |
| *Setting* | | | |
| Setting of data collection | 14 | Where was the data collected? e.g. home, clinic, workplace | 4 |
| Presence of non-participants | 15 | Was anyone else present besides the participants and researchers? | 4 |
| Description of sample | 16 | What are the important characteristics of the sample? e.g. demographic data, date | 5 |
| *Data collection* | | | |
| Interview guide | 17 | Were questions, prompts, guides provided by the authors? Was it pilot tested? | 4 |
| Repeat interviews | 18 | Were repeat inter views carried out? If yes, how many? | N/A |
| Audio/visual recording | 19 | Did the research use audio or visual recording to collect the data? | 4 |
| Field notes | 20 | Were field notes made during and/or after the inter view or focus group? | N/A |
| Duration | 21 | What was the duration of the inter views or focus group? | 4 |
| Data saturation | 22 | Was data saturation discussed? | 14 |
| Transcripts returned | 23 | Were transcripts returned to participants for comment and/or | N/A |

| Topic | Item No. | Guide Questions/Description | Reported on Page No. |
|---|---|---|---|
| | | correction? | |
| **Domain 3: analysis and findings** | | | |
| *Data analysis* | | | |
| Number of data coders | 24 | How many data coders coded the data? | 4 |
| Description of the coding tree | 25 | Did authors provide a description of the coding tree? | N/A |
| Derivation of themes | 26 | Were themes identified in advance or derived from the data? | 4 |
| Software | 27 | What software, if applicable, was used to manage the data? | 4 |
| Participant checking | 28 | Did participants provide feedback on the findings? | N/A |
| *Reporting* | | | |
| Quotations presented | 29 | Were participant quotations presented to illustrate the themes/findings? Was each quotation identified? e.g. participant number | 8 to 12 |
| Data and findings consistent | 30 | Was there consistency between the data presented and the findings? | Yes |
| Clarity of major themes | 31 | Were major themes clearly presented in the findings? | Yes |
| Clarity of minor themes | 32 | Is there a description of diverse cases or discussion of minor themes? | No |

Developed from: Tong A, Sainsbury P, Craig J. Consolidated criteria for reporting qualitative research (COREQ): a 32-item checklist for interviews and focus groups. *International Journal for Quality in Health Care*. 2007. Volume 19, Number 6: pp. 349 – 357

## Characteristics of interviewees (Tables S1-S4)

11 of the 41 participants agreed to be interviewed. Characteristics of interviewees (n = 11) are tabulated below (Tables S1-S3), and nutritional intakes summarised in Table S4. Profiles and nutritional intakes of interviewees are reflective of the full dataset.

Table S1. Demographic profile of interviewees (n = 11).

| **Characteristics** | **Mean ± SD or n (%)** |
|---|---|
| Age at interview (years) | 33.0 ± 5.8 |
| Ethnicity | |
| Chinese | 2 (18.2) |
| Malay | 7 (63.6) |
| Indian | 0 (0.0) |
| Others | 2 (18.2) |
| Highest education level | |
| Primary | 0 (0.0) |
| Secondary | 5 (45.5) |
| Post-Secondary | 6 (54.5) |
| Employment status | |
| Unemployed | 10 (90.9) |
| Employed | 1 (9.1) |
| Average household income (SGD) per month | |
| Less than $500 | 9 (81.8) |
| $500-1,000 | 2 (18.2) |
| Smoking status during pregnancy | |
| Smoker | 3 (27.3) |
| Non-smoker | 8 (72.7) |
| Ever consumed alcohol during pregnancy | |
| No | 10 (90.9) |
| Yes | 1 (9.1) |

Table S2. Gestational characteristics and anthropometric measurements of interviewees (n = 11).

| **Characteristic** | **Mean ± SD or n (%)** |
|---|---|
| Gestational age at point of interview (weeks) | 36.4 ± 1.5 |
| Gestational age at first doctor's visit (weeks) | 10.0 ± 5.5 |
| Number of children | |
| 0 | 1 (9.1) |
| 1 | 1 (9.1) |
| 2 | 4 (36.4) |
| ≥3 | 5 (45.5) |
| Planned pregnancy | |
| No | 7 (63.6) |
| Yes | 4 (36.4) |
| Diagnosed with gestational diabetes | |
| No | 6 (54.4) |
| Yes | 2 (18.2) |
| Not reported / known | 3 (27.3) |
| Diagnosed with other health problems | |
| No | 9 (81.8) |
| Yes | 2 (18.2) |
| Supplement intake during pregnancy | |
| Folic acid | 9 (81.8) |
| Iron | 4 (36.4) |
| Calcium | 1 (9.1) |
| Vitamin D | 1 (9.1) |
| Multivitamin | 10 (90.9) |
| Fish oil | 5 (45.5) |
| Pre-pregnancy body mass index (BMI) ($kg/m^2$) based on reported weight | 26.6 ± 10.3 |
| <18.5 | 2 (18.2) |
| 18.5 – 24.9 | 4 (36.4) |

| | |
|---|---|
| 25.0 – 29.9 | 1 (9.1) |
| ≥30.0 | 3 (27.3) |
| Not reported / known | 1 (9.1) |
| Blood pressure (mmHg) | |
| Systolic | 109.3 ± 13.0 |
| Diastolic | 69.5 ± 8.9 |

Table S3. Biomarker profile of interviewees.

| **Biomarkers** | **n** | **Normal readings (%)** | **Mean ± SD** | **Reference values** |
|---|---|---|---|---|
| Haemoglobin (g/dL) | 11 | 9.1 | 11.0 ± 0.8 | 12.0 – 16.0 |
| 25-Hydroxyvitamin D (ng/mL) | 11 | 63.6 | 20.8 ± 8.3 | 20.0 – 100.0 |
| Fasting plasma glucose (mmol/L) | 7 | 100.0 | 4.2 ± 0.7 | ≤ 5.0* |

*Reference value specific to pregnancy.

Table S4. Mean nutrient intake with reference to daily recommendations during pregnancy (n = 11).

| **24-Hour recall data** | **Mean ± SD** | **Recommendations** |
|---|---|---|
| Energy intake (kcal) | 1571.6 ± 517.6 | 1800-2250 kcal/day [a] |
| Macronutrients | | |
| Protein intake (g) | 66.1 ± 34.7 | 71 g/day [b] |
| % kcal from protein | 16.1 ± 4.7 | 10 – 35% [c] |
| Total fat (g) | 51.1 ± 19.4 | N.A. |
| % kcal from total fat | 29.8 ± 9.0 | 20 – 35% [c] |
| Saturated fat (g) | 19.0 ± 10.2 | N.A. |
| % kcal from saturated fat | 10.8 ± 5.7 | < 10% [c] |
| Carbohydrate (g) | 208.0 ± 78.1 | 175 g/day [b] |
| % kcal from carbohydrates | 53.2 ± 10.2 | 45 – 65% [c] |
| Dietary fibre (g) | 11.6 ± 7.6 | 28 g/day [b] |
| Added sugar (g) | 59.4 ± 52.1 | N.A. |
| % kcal from sugar | 15.4 ± 13.4 | <10% [c] |
| Servings of major food groups | | |
| Grains | 7.0 ± 5.8 | 6-7 [a] |
| Fruit | 0.4 ± 0.8 | 2 [a] |
| Vegetables | 0.5 ± 0.6 | 3 [a] |
| Protein | 1.2 ± 1.0 | 2.5 [a] |
| Dairy products | 0.5 ± 0.5 | 1 [a] |
| Micronutrients (minerals) (mg) | | |
| Calcium | 620.3 ± 399.3 | 1000 [a] |
| Zinc | 7.4 ± 4.1 | 10 [a] |
| Iron | 8.1 ± 4.6 | 27 [a] |
| Sodium | 2234.5 ± 1219.3 | 2000 [a] |

[a] Nutritional recommendations during pregnancy available from Ministry of Health, Singapore (HealthHub) [11]

[b] Recommended quantity of macronutrients during pregnancy [12]

[c] Recommendations based on the acceptable macronutrient distribution range [10]

## Supplementary Quotes S1 (Theme 1)

Supplementary quotes that illustrate food management practices due to financial constraints are tabulated below. The categories describe the subtheme of 'Coping with limited resources'.

| **Categories** | **Illustrative quotes** |
|---|---|
| Cost as a limiting factor to choice of food and variety | *"Because we tried to find the fresh one (prawns) at the supermarket. It's so expensive! I'm like, never mind, just buy the frozen one. It's like $10 cheaper."*<br>*(PW024, 36 weeks)* |
| Comparison of product prices between supermarkets to manage budget | *"Previously when I got salary, I will buy like Sheng Siong, I will spend onto at Sheng Siong rather than at Prime. Because Sheng Siong a bit cheaper compared to Prime supermarket. Prime quite expensive."*<br>*(PW026, 39 weeks)* |
| Careful portioning of cooked meals | *"Like see one person can get how many piece. ...If I cook a lot, they find that the food is not nice, then wasted, throw. So I always cook like counting only."*<br>*(PW008, 38 weeks)* |
| Fruits and vegetables less of a priority | *"I don't encourage them (family) to buy and then it's sitting in the fridge and then they…they waste it. They will eat on the day they buy, and then the balance nobody eats. And then when I open the fridge it's like, oh my god, it's rotten. Then you waste the money. Then fruits not cheap leh."*<br>*(PW015, 36 weeks)*<br>*"No money right, skip the fruits, vegetables. Sometimes also never cook vegetables, just cook the meal…the meat only lah."*<br>*(PW036, 35 weeks)* |
| Unpredictability of having sufficient food | *"Sometimes you know like, when if something happened this month, then we have to cut out groceries for the following month. Especially like, if my like, recently my husband went through surgery he was not working for two months."*<br>*(PW029, 35 weeks)* |
| One type of meal, or only one meal, for the day | *"We make it like, like how to say? Make sure that the food can be last for. So at times, one day twice, we eat. Or at times, one day once."*<br>*(PW026, weeks)*<br>*"Like currently, usually, we usually eat like easily…at most once, once a day. Usual one lah, it's usually once a day."*<br>*(PW032, 36 weeks)* |
